# Comparison of Artemether Versus Quinine for the Treatment of Cerebral Malaria in Children: A Meta-Analysis of Randomized Controlled Trials

**DOI:** 10.7759/cureus.91244

**Published:** 2025-08-29

**Authors:** Hania Saeed, Danyal Tahir, Muhammad Muneebullah Khan, Mohammad Taimoor Faheem, Ayesha H Malik, Samir Saeed, Sharjeel Azeem, Mikram Syed, Abbas Allami

**Affiliations:** 1 Internal Medicine, Dow University of Health Sciences, Dow International Medical College, Karachi, PAK; 2 Medicine, Dow University of Health Sciences, Dow International Medical College, Karachi, PAK; 3 Medicine and Surgery, Dow University of Health Sciences, Dow International Medical College, Karachi, PAK; 4 Infectious Diseases, Qazvin University of Medical Sciences, Qazvin, IRN

**Keywords:** artemether, cerebral malaria, malaria, meta-analysis, quinine

## Abstract

Malaria is a potentially life-threatening parasitic disease caused by a protozoal infection via Plasmodium species, transmitted by a carrier female Anopheles mosquito. Cerebral malaria is typically caused by Plasmodium falciparum and is known as a fatal neurological complication of malaria.

This systematic review and meta-analysis was performed due to limited research on the comparison of artemether and quinine for the treatment of cerebral malaria in children. Several electronic databases (PubMed, Cochrane Library, and Scopus) were searched from inception to February 2023. Additionally, risk of bias and quality assessment were performed using the Cochrane Risk of Bias Tool for Randomized Controlled Trials. A total of 7 studies were pooled, including 618 patients receiving artemether and 607 patients receiving quinine, leading to a pooling of 1,225 patients in this meta-analysis. It was observed that parasite clearance time (mean difference (MD): -7.63; 95% confidence interval (CI): -11.06, -4.21, P < 0.0001) was significantly improved in the artemether cohort. However, fever clearance time, coma clearance time, and mortality rate failed to reach statistically significant differences.

In summary, artemether demonstrated a mildly superior efficacy in comparison to quinine. Thus, regions with artemisinin-sensitive strains of malaria are encouraged to continue the use of artemisinin; however, regions with artemisinin resistance may consider the use of quinine as an alternative in the treatment of cerebral malaria in children. Future comprehensive randomized controlled studies are needed to arrive at a robust conclusion.

## Introduction and background

Malaria is a potentially life-threatening parasitic disease caused by a protozoal infection via Plasmodium species, transmitted by a carrier female Anopheles mosquito. Typical symptoms of malaria include high fevers, chills, muscle aches, and fatigue. Cerebral malaria, typically caused by Plasmodium falciparum infection, is a fatal neurological complication of malaria. If not treated in a timely manner, cerebral malaria can progress to coma or death [1]. Cerebral malaria is most commonly diagnosed in Sub-Saharan Africa, where it affects about 1% of children below the age of 5, and alone accounts for approximately 13% of malaria-related deaths [2]. The pathophysiology of cerebral malaria is due to the damaged vascular endothelium caused by parasite sequestration, inflammatory cytokine production, and vascular leakage, which leads to brain hypoxia, as indicated by increased levels of alanine and lactate [3]. Clinical manifestations of cerebral malaria may include diffuse encephalopathy with a history of fever for up to three days, subsequent seizures, and loss of consciousness. The clinical hallmark of cerebral malaria is coma, which is thought to be due to parasitized red blood cells (RBCs) sequestered in cerebral microcirculation.

Malaria can be effectively treated by either quinine or artemether, a drug derived from artemisinin. Artemether is quickly absorbed by the body and then metabolized into dihydroartemisinin. It has a relatively short half-life of about an hour and is a highly effective anti-malarial drug that works by swiftly reducing the number of parasites in the body, leading to a rapid improvement in symptoms. The absorption of artemether is greatly influenced by co-administration with fat and thus improves significantly as the patient recovers from malaria [4]. The drug preceding artemether is quinine, an alkaloid drug extracted from Cinchona bark, first discovered in the nineteenth century. Quinine can inhibit Plasmodium falciparum's nucleic acid synthesis, protein synthesis, and glycolysis, as well as bind with hemazoin in parasitized erythrocytes to interfere with the parasite's ability to digest hemoglobin. Furthermore, it can prevent the spontaneous formation of beta-hematin, which is a toxic product of the digestion of hemoglobin by parasites. It is commonly used as chemoprophylaxis in certain regions of the world, particularly those with artemisinin-resistant strains of the parasite.

Treatment of cerebral malaria traditionally involves a handful of conventional anti-malarial drugs available on the market, such as quinine or artemisinins, as well as supportive measures like stabilizing the patient initially, blood transfusions, using osmotic diuretics, correcting hypoglycemia, acidosis, and hypovolemia, and employing immunomodulation [5]. While providing general care is essential, it is also necessary to standardize supportive care for patients with cerebral malaria [6]. The administration of intravenous injections of artesunate is considered to be the preferred treatment for cerebral malaria. Artesunate is one of the derivatives of artemisinin, similar to artemether. However, in certain regions of the world, artemether is still widely used due to the low availability of artesunate, such as in Equatorial Guinea. Therefore, we conducted a systematic review and meta-analysis comparing the efficacy of artemether versus quinine for the treatment of cerebral malaria in children, with the addition of a pre-specified subgroup analysis exploring the effectiveness of various methods of administering the drugs, including intravenous, intramuscular, and rectal administration.

## Review

Methodology 

Data Sources and Search Strategy

This meta-analysis was performed as per Preferred Reporting Items for Systematic Review and Meta-Analyses (PRISMA) guidelines [7]. Databases including PubMed, Cochrane Library, and Scopus were used for the systematic search, from inception to February 2023. The comprehensive search strategy employed for each database is available in Table 1.

**Table 1:** Search strategy used for the systematic review

SEARCH STRATEGY	DATABASE	CITATIONS
("artemether"[MeSH Terms] OR "artemether"[All Fields] OR (("artemisinin"[Supplementary Concept] OR "artemisinin"[All Fields] OR "artemisinine"[All Fields] OR "artemisinins"[MeSH Terms] OR "artemisinins"[All Fields] OR "artemisinin s"[All Fields]) AND ("analogs and derivatives"[MeSH Subheading] OR ("analogs"[All Fields] AND "derivatives"[All Fields]) OR "analogs and derivatives"[All Fields] OR "derivatives"[All Fields] OR "derivable"[All Fields] OR "derivant"[All Fields] OR "derivants"[All Fields] OR "derivate"[All Fields] OR "derivated"[All Fields] OR "derivates"[All Fields] OR "derivation"[All Fields] OR "derivations"[All Fields] OR "derivative"[All Fields] OR "derive"[All Fields] OR "derived"[All Fields] OR "derives"[All Fields] OR "deriving"[All Fields])) OR (("inject"[All Fields] OR "injectability"[All Fields] OR "injectant"[All Fields] OR "injectants"[All Fields] OR "injectate"[All Fields] OR "injectates"[All Fields] OR "injected"[All Fields] OR "injectible"[All Fields] OR "injectibles"[All Fields] OR "injecting"[All Fields] OR "injections"[MeSH Terms] OR "injections"[All Fields] OR "injectable"[All Fields] OR "injectables"[All Fields] OR "injection"[All Fields] OR "injects"[All Fields]) AND ("artemether"[MeSH Terms] OR "artemether"[All Fields]))) AND ("quinine"[MeSH Terms] OR "quinine"[All Fields] OR "quinines"[All Fields] OR (("intraveneous"[All Fields] OR "intraveneously"[All Fields] OR "intravenous"[All Fields] OR "intravenously"[All Fields]) AND ("quinine"[MeSH Terms] OR "quinine"[All Fields] OR "quinines"[All Fields]))) AND ("malaria"[MeSH Terms] OR "malaria"[All Fields] OR "malarias"[All Fields] OR "malaria s"[All Fields] OR "malariae"[All Fields] OR (("complicances"[All Fields] OR "complicate"[All Fields] OR "complicated"[All Fields] OR "complicates"[All Fields] OR "complicating"[All Fields] OR "complication"[All Fields] OR "complication s"[All Fields] OR "complications"[MeSH Subheading] OR "complications"[All Fields]) AND ("malaria"[MeSH Terms] OR "malaria"[All Fields] OR "malarias"[All Fields] OR "malaria s"[All Fields] OR "malariae"[All Fields])) OR ("malaria, cerebral"[MeSH Terms] OR ("malaria"[All Fields] AND "cerebral"[All Fields]) OR "cerebral malaria"[All Fields] OR ("cerebral"[All Fields] AND "malaria"[All Fields])) OR (("neurologic"[All Fields] OR "neurological"[All Fields] OR "neurologically"[All Fields]) AND ("malaria"[MeSH Terms] OR "malaria"[All Fields] OR "malarias"[All Fields] OR "malaria s"[All Fields] OR "malariae"[All Fields]))) AND ("child"[MeSH Terms] OR "child"[All Fields] OR "children"[All Fields] OR "child s"[All Fields] OR "children s"[All Fields] OR "childrens"[All Fields] OR "childs"[All Fields] OR ("adolescences"[All Fields] OR "adolescency"[All Fields] OR "adolescent"[MeSH Terms] OR "adolescent"[All Fields] OR "adolescence"[All Fields] OR "adolescents"[All Fields] OR "adolescent s"[All Fields]))	PUBMED	215
(Artemether OR Artemisinin derivative OR P01BE02 OR Injectable artemether) AND (Quinine OR M09AA01 OR P01BCO1 OR Intravenous quinine) AND (Malaria OR Complicated malaria OR Cerebral malaria OR Neurological malaria) AND (Children OR Adolescents OR Infant)	COCHRANE LIBRARY	112
TITLE-ABS-KEY ((“Artemether” OR “Artemisinin derivative” OR “P01BE02” OR “Injectable artemether”) AND (“Quinine” OR “M09AA01” OR “P01BCO1” OR “Intravenous quinine”) AND (“Malaria” OR “Complicated malaria” OR “Cerebral malaria” OR “Neurological malaria”) AND (“Children OR Adolescents”)	SCOPUS	72

The methodology of this study has been rigorously assessed using the Assessing the Methodological Quality of Systematic Reviews (AMSTAR-2) guidelines [8].

Study Selection and Eligibility Criteria

All articles retrieved from the systematic search were exported to the EndNote reference library, version X8.1 (Clarivate Analytics), followed by the removal of duplicates. Articles exclusively following the pre-specified eligibility criteria were included. The pre-specified eligibility criteria included: (i) published and completed randomized controlled trials, (ii) studies comparing artemether with quinine, (iii) patients <18 years of age suffering from cerebral malaria, and (iv) studies reporting at least one of the pre-selected outcomes. Case reports, letters, and systematic reviews were excluded during the screening process.

Data Extraction and Quality of Assessment

The following data were extracted from the included studies: (a) patient baseline characteristics, (b) fever clearance time, (c) parasite clearance time, (d) coma recovery time, and (e) rate of mortality. Quality assessment was performed in conformity with the Cochrane Risk of Bias Tool for randomized controlled trials [9].

Statistical Analysis

Review Manager (RevMan version 5.3; Copenhagen: The Nordic Cochrane Centre, The Cochrane Collaboration, 2014) was used for statistical analyses, with the random-effects model employed in order to generate our results [10]. Statistical significance was determined via the use of p-values, which were considered statistically significant when p < 0.05. In outcomes demonstrating p-values at 0.05 or above were declared statistically insignificant. In this analysis, the continuous outcomes were compared using mean differences, whereas the dichotomous outcomes were compared using risk ratios. Evaluation for the degree of heterogeneity of included studies was performed via the Higgins I2 statistic value, with values under 25% demonstrating low heterogeneity, values under 75% demonstrating moderate heterogeneity, and values at 75% or above demonstrating high heterogeneity [11].

Results

Literature Search, Quality Assessment, and Baseline Characteristics

**Figure 1:**
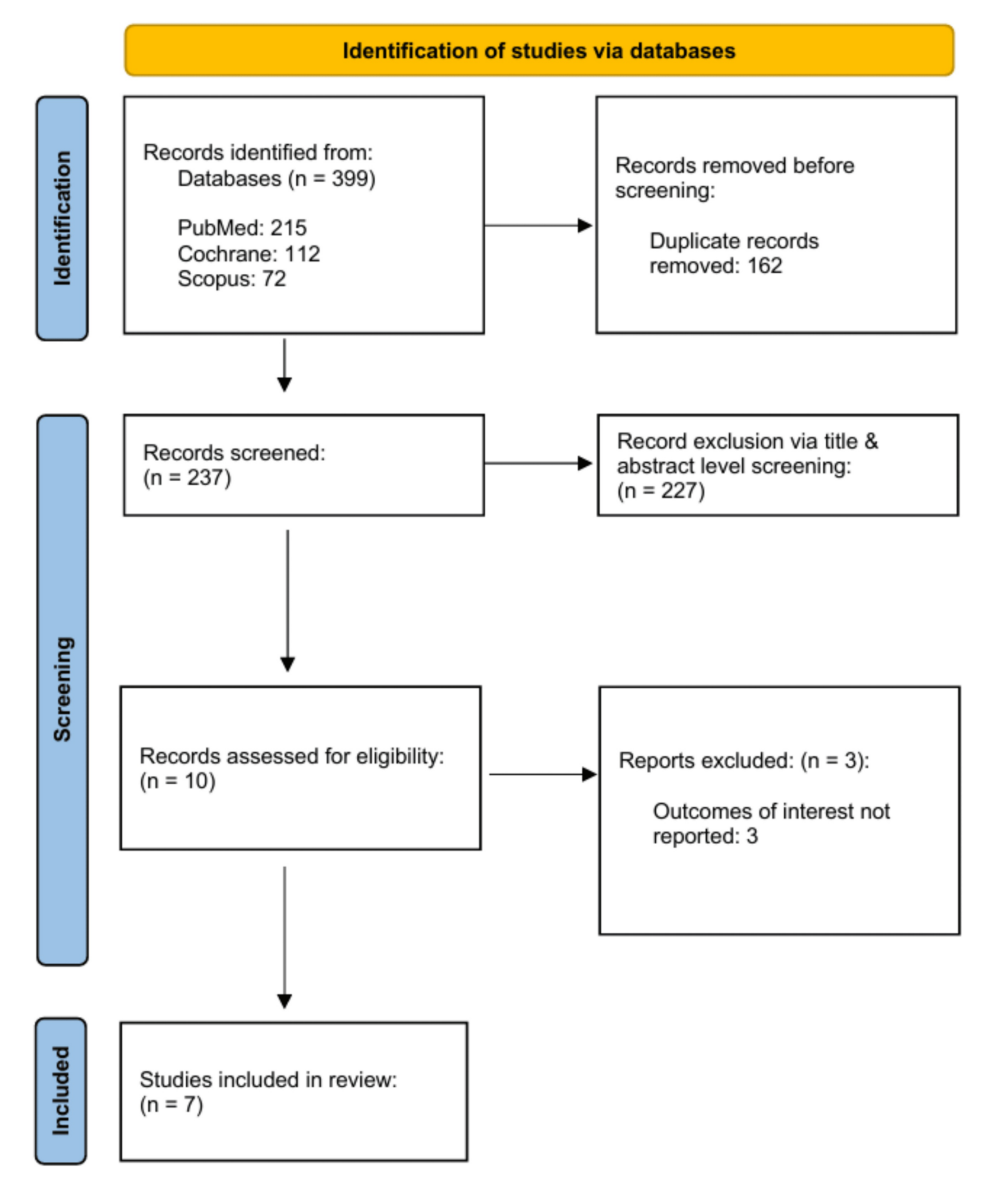
PRISMA flowchart PRISMA: Preferred Reporting Items for Systematic Review and Meta-Analyses

The initial search generated 399 relevant articles up to February 2023. After the removal of duplicate studies, with the conduction of a comprehensive screening process, there were seven articles selected for inclusion within this systematic review and meta-analysis [12-18]. The details of the screening process are outlined in the PRISMA flowchart, as illustrated in Figure 1.

**Figure 2:**
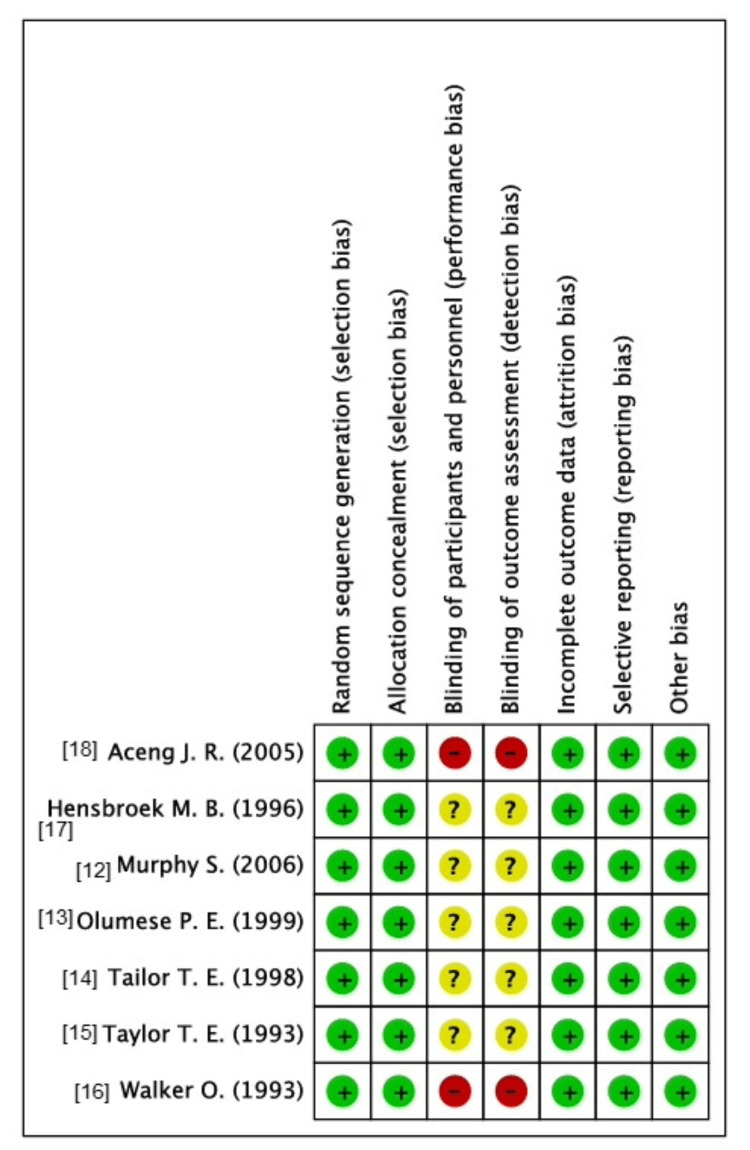
Summary of risk of bias assessment using the Cochrane Risk of Bias tool for RCTs RCT: randomized controlled trial

The quality assessment was performed in conformity with the Cochrane Risk of Bias Tool for randomized controlled trials (RCTs) [9]. Overall, the included studies were deemed to be of moderate quality and demonstrated an overall low-to-moderate risk of bias, summarized in Figure 2.

The comprehensive judgements of each of the included studies are available in Table 2. 

**Table 2:** Detailed assessments of the Cochrane Risk of Bias tool for RCTs RCT: randomized controlled trial

Study Title	Bias	Rating	Author Judgment
Murphy et al. [12]	Random sequence generation (selection bias)	Low Risk	Patients were randomized.
	Allocation concealment (selection bias)	Low Risk	Random allocation was conducted via a sealed envelope.
	Blinding of participants and personnel (performance bias)	Unclear Risk	There was no information provided regarding blinding.
	Blinding of outcome assessment (detection bias)	Unclear Risk	There was no information provided regarding blinding.
	Incomplete outcome data (attrition bias)	Low Risk	The exclusion or loss of patients was explained in the study and are unlikely to cause bias.
	Selective reporting (reporting bias)	Low Risk	All pre-specified outcomes were reported.
	Other bias	Low Risk	The study appears to be free from any other sources of bias.
Olumese et al. [13]	Random sequence generation (selection bias)	Low Risk	Patients were randomized.
	Allocation concealment (selection bias)	Low Risk	Random allocation was conducted.
	Blinding of participants and personnel (performance bias)	Unclear Risk	There was no information provided regarding blinding.
	Blinding of outcome assessment (detection bias)	Unclear Risk	There was no information provided regarding blinding
	Incomplete outcome data (attrition bias)	Low Risk	The exclusion or loss of patients was explained in the study and is unlikely to cause bias.
	Selective reporting (reporting bias)	Low Risk	All prespecified outcomes were reported
	Other bias	Low Risk	The study appears to be free from any other source of bias.
Taylor et al 1998. [14]	Random sequence generation (selection bias)	Low Risk	Patients were randomized.
	Allocation concealment (selection bias)	Low Risk	Random allocation was conducted.
	Blinding of participants and personnel (performance bias)	Unclear Risk	There was no information provided regarding blinding.
	Blinding of outcome assessment (detection bias)	Unclear Risk	There was no information provided regarding blinding.
	Incomplete outcome data (attrition bias)	Low Risk	The exclusion or loss of patients was explained in the study and is unlikely to cause bias.
	Selective reporting (reporting bias)	Low Risk	All prespecified outcomes were reported.
	Other bias	Low Risk	The study appears to be free from any other sources of bias.
Taylor et al 1993. [15]	Random sequence generation (selection bias)	Low Risk	Patients were randomized.
	Allocation concealment (selection bias)	Low Risk	Random allocation was conducted.
	Blinding of participants and personnel (performance bias)	Unclear Risk	There was no information provided regarding blinding.
	Blinding of outcome assessment (detection bias)	Unclear Risk	There was no information provided regarding blinding.
	Incomplete outcome data (attrition bias)	Low Risk	The exclusion or loss of patients was explained in the study and are unlikely to cause bias.
	Selective reporting (reporting bias)	Low Risk	All pre-specified outcomes were reported.
	Other bias	Low Risk	The study appears to be free from any other sources of bias.
Walker et al. [16]	Random sequence generation (selection bias)	Low Risk	Patients were randomized.
	Allocation concealment (selection bias)	Low Risk	Random allocation was conducted via number sequence generation.
	Blinding of participants and personnel (performance bias)	High Risk	This study did not include any blinding procedures.
	Blinding of outcome assessment (detection bias)	High Risk	This study did not include any blinding procedures.
	Incomplete outcome data (attrition bias)	Low Risk	The exclusion or loss of patients was explained in the study and are unlikely to cause bias.
	Selective reporting (reporting bias)	Low Risk	All pre-specified outcomes were reported.
	Other bias	Low Risk	The study appears to be free from any other sources of bias.
Hensbroek et al. [17]	Random sequence generation (selection bias)	Low Risk	Patients were randomized.
	Allocation concealment (selection bias)	Low Risk	Random allocation was conducted.
	Blinding of participants and personnel (performance bias)	Unclear Risk	There was no information provided regarding blinding.
	Blinding of outcome assessment (detection bias)	Unclear Risk	There was no information regarding blinding.
	Incomplete outcome data (attrition bias)	Low Risk	The exclusion or loss of patients was explained in the study and accounted for.
	Selective reporting (reporting bias)	Low Risk	All pre-specified outcomes were reported.
	Other bias	Low Risk	There appear to be no other sources of bias in this study.
Aceng et al. [18]	Random sequence generation (selection bias)	Low Risk	Patients were randomized.
	Allocation concealment (selection bias)	Low Risk	Random allocation was conducted.
	Blinding of participants and personnel (performance bias)	High Risk	No blinding procedures were carried out.
	Blinding of outcome assessment (detection bias)	High Risk	There was no information provided regarding blinding.
	Incomplete outcome data (attrition bias)	Low Risk	There was no exclusion or loss of patients.
	Selective reporting (reporting bias)	Low Risk	All prespecified outcomes were reported.
	Other bias	Low Risk	There appear to be no other sources of bias in this study.

The baseline characteristics of the included study populations are outlined in Table 3.

**Table 3:** Baseline characteristics of included studies

Study	Country	Receiving Artemether, n	Receiving Quinine, n	Dosing Strategy			Mode of Administration			Mean Age, years			Male, n		Hemoglobin, g/dL		Duration of Coma, h		Hypoglycemia, %			Temperature, °C	
				Artemether		Quinine	Artemether		Quinine	Artemether		Quinine	Artemether	Quinine	Artemether	Quinine	Artemether	Quinine	Artemether		Quinine	Artemether	Quinine
Murphy et al. [12]	Kenya	89	71	1.6 mg/kg daily	20 mg/kg loading dose, followed by 10 mg/kg every 8 hours		IM	IV		2.2 (0.4-9)	2.5 (0.4-12)		44	36	7.2 (2.8-14)	7.3 (2.9-11.9)	10 (0-72)	7 (0-72)	32.5	21.5		-	-
Olumese et al. [13]	Nigeria	54	49	3.2 mg/kg loading dose, followed by 1.6 mg/kg daily	20 mg/kg loading dose, followed by 10 mg/kg every 8 hours		IM	IV		3.1 (1-5)	3.2 (1-5)		-	-	-	-	-	-	12	4		38.5	38.6
Taylor et al. [14]	Malawi	83	81	3.2 mg/kg loading dose, followed by 1.6 mg/kg daily	20 mg/kg over 4 hours		IM	IV		2.9 (1-4.9)	3.2 (1.2-5.2)		44	39	8.1 (2.2)	8.2 (2.5)	-	-	13	11		38.9 (1.12)	38.9 (1.25)
Taylor et al. [15]	Malawi	28	37	3.2 mg/kg loading dose, followed by 1.6 mg/kg daily	20 mg/kg loading dose, followed by 10 mg/kg every 8 hours		IM	IV		2.9 (1-7)	2.8 (1-7)		14	17	-	-	3.7 (4.0)	4.6 (3.8)	829	1150		39.0 (1.0)	39.1 (1.2)
Walker et al. [16]	Nigeria	25	29	3.2 mg/kg loading dose, followed by 1.6 mg/kg daily	20 mg/kg loading dose, followed by 10 mg/kg every 8 hours		IM	IV		3 (1.7-4.3)	3 (1.9-4.1)		-	-	-	-	-	-	-	-		-	-
Hensbroek et al. [17]	Gambia	288	288	3.2 mg/kg loading dose, followed by 1.6 mg/kg daily	20 mg/kg loading dose, followed by 10 mg/kg every 12 hours		IM	IM		4 (2.2-5.8)	3.8 (2-5.6)		152	143	8.1 (2.6)	8.4 (2.5)	6.0 (1-150)	6.0 (1-96)	26.3	22.3		38.8 (1.1)	38.8 (1.0)
Aceng et al. [18]	Uganda	51	52	120 mg loading dose, followed by 80 mg daily	20 mg/kg loading dose, followed by 10 mg/kg every 8 hours		Rectal	IV		2.3 (2.1-3.5)	2.2 (0.8-3.6)		-	-	1.6 (0.5)	1.4 (0.6)	11.8 (9.6)	11.3 (10.3)	-	-		38.3 (1.3)	38.9 (0.9)

Fever Clearance Time

**Figure 3:**
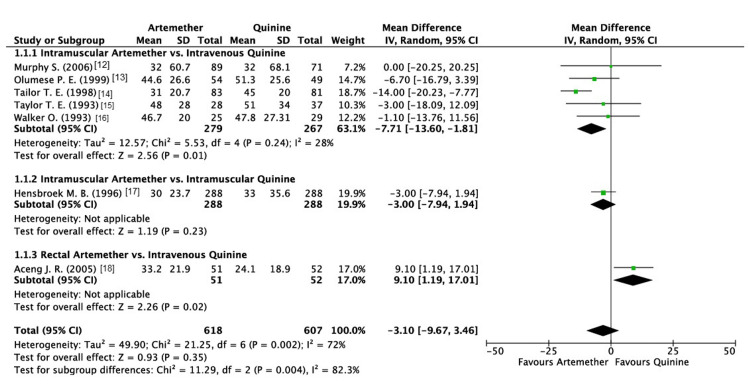
Forest plot of fever clearance time

The fever clearance time (in hours) was reported by all seven of the included studies. The analysis revealed that artemether did not make a significant difference to fever clearance time as compared to quinine (mean difference (MD): -3.10; 95% confidence interval (CI): -9.67, 3.46; P = 0.35; I2 = 72%) (Figure 3).

With respect to the subgroup analysis performed, it displayed a significant difference between various methods of administration (P = 0.004; I2 = 82.3%; Figure 3). Intramuscular (IM) administration of artemether demonstrated greater efficacy as compared to intravenous(IV) quinine. On the other hand, rectal administration of artemether demonstrated poorer results when compared to IV quinine (P = 0.02). Contrastingly, no significant differences were noted when IM artemether was compared to IM quinine (P = 0.23).

**Figure 4:**
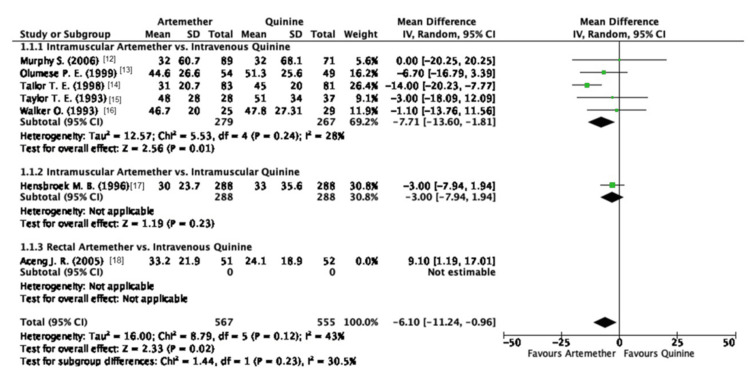
Forest plot of fever clearance time (HR) after sensitivity analysis

Due to the high heterogeneity observed in this outcome, a sensitivity analysis was performed using the leave-one-out method. The study conducted by Aceng et al. was omitted [18], which resulted in a reduction of heterogeneity and highlighted the superiority of artemether when compared to quinine, which differs from the original finding of the non-significant difference between artemether and quinine (MD: -6.10; 95% CI: -11.24, -0.96) (Figure 4).

There was a reduction in the overall heterogeneity; however, it could not be completely eradicated (I2 = 43%; P = 0.12).

Parasite Clearance Time

**Figure 5:**
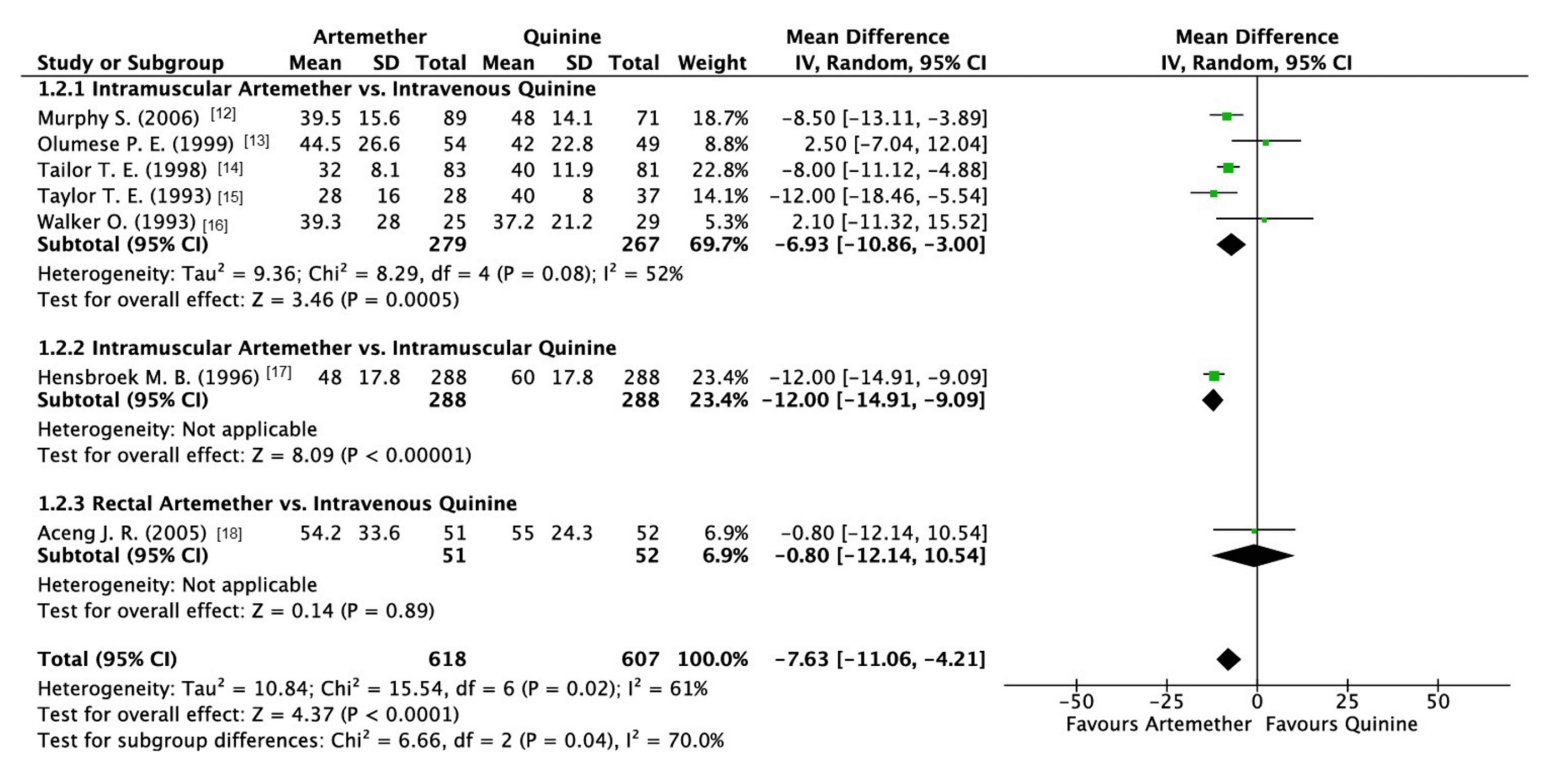
Forest plot of parasite clearance time

The parasite clearance time (in hours) was reported by seven studies. The analysis revealed that artemether significantly improved parasite clearance time as compared to quinine (MD: -7.63; 95% CI: -11.06, -4.21, P < 0.0001; I2 = 61%) (Figure 5). 

With respect to the subgroup analysis performed, it displayed a significant difference between various methods of administration (P = 0.04; I2 = 82.3%; Figure 5). IM administration of artemether demonstrated greater efficacy when compared to IV and IM quinine (P = 0.005, P < 0.00001). Contrastingly, no significant differences were noted when rectal artemether was compared to IV quinine (P = 0.89).

**Figure 6:**
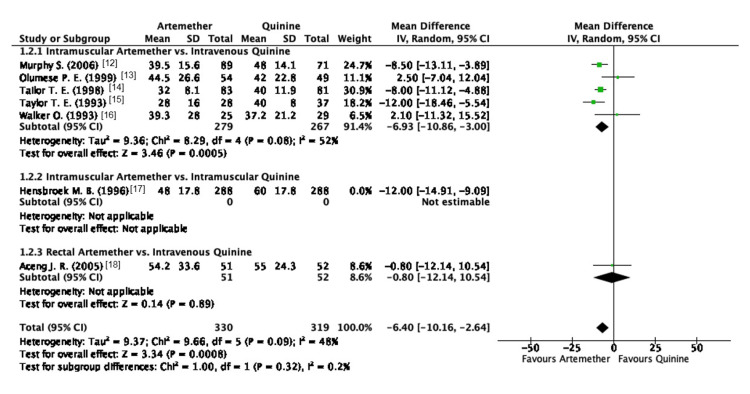
Forest plot of parasite clearance time (hr) after sensitivity analysis

Due to the high heterogeneity observed in this outcome, a sensitivity analysis was performed using the leave-one-out method. The study conducted by Hensbroek et al. was omitted [17], which resulted in a reduction of heterogeneity. There was a reduction in the overall heterogeneity; however, it could not be completely eradicated (I2 = 48%; P = 0.09) (Figure 6). 

Coma Clearance Time

**Figure 7:**
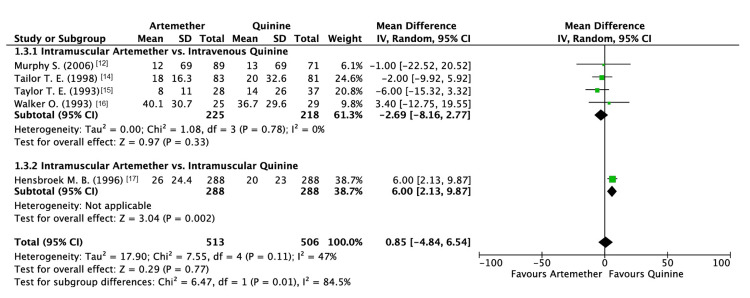
Forest plot of coma clearance time

Coma clearance time (in hours) was reported by five studies. The analysis revealed that artemether did not have a significant difference in coma clearance time as compared to quinine (MD: 0.85; 95% CI: -4.84, 6.54, P = 0.77) (Figure 7).

With respect to the subgroup analysis performed, it displayed a significant difference between various methods of administration (P = 0.01; I2 = 84.5%; Figure 7). IM administration of artemether did not demonstrate greater efficacy when compared to IV quinine (P = 0.78). Contrastingly, IM quinine demonstrated a significant difference as compared to IM artemether (P = 0.002).

Mortality Rate

**Figure 8:**
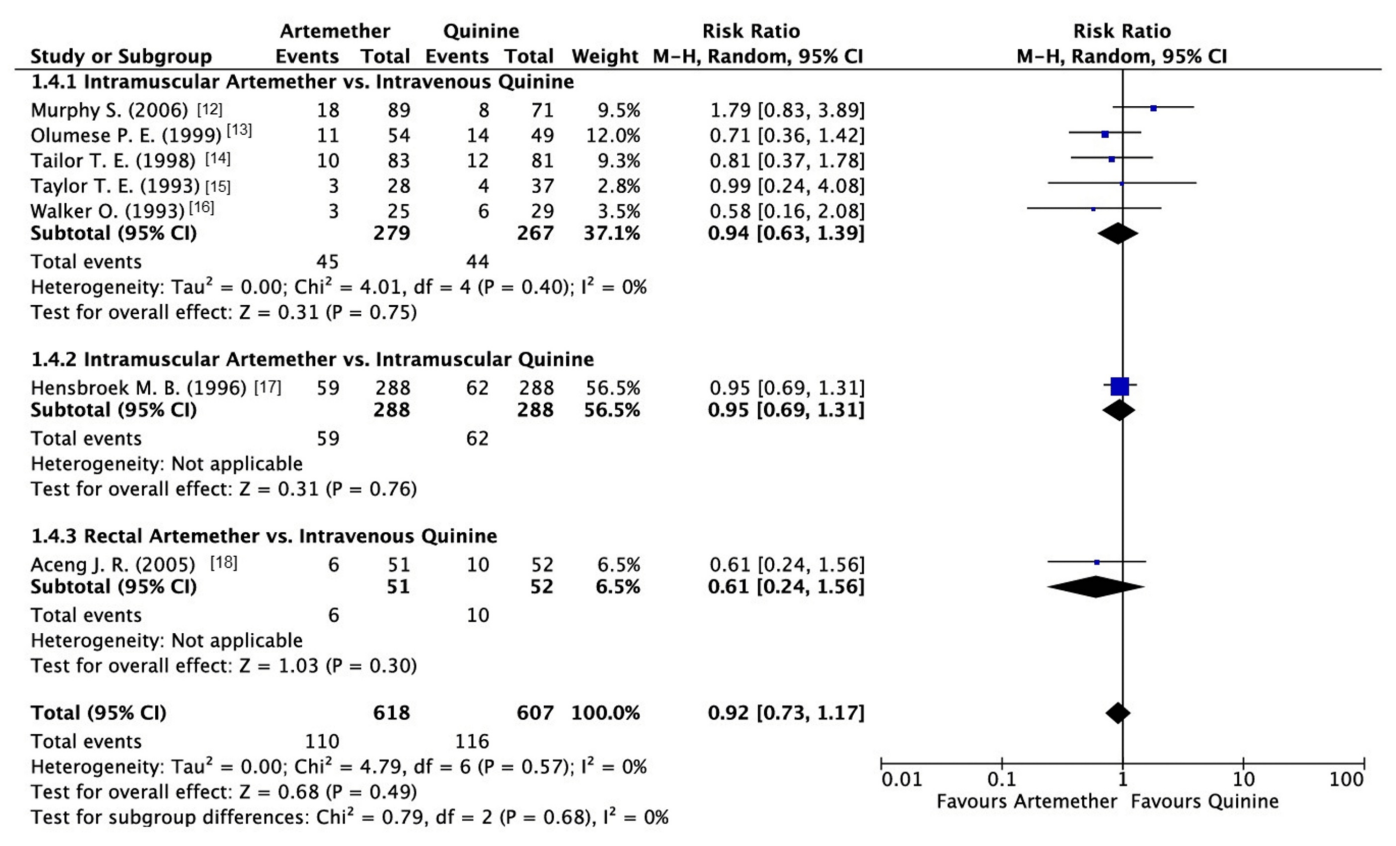
Forest plot of mortality

The mortality rate was reported by seven studies. The analysis revealed that artemether did not have a significant difference in mortality rate as compared to quinine (RR: 0.92; 95% CI: 0.73, 1.17, P = 0.49) (Figure 8).

With respect to the subgroup analysis performed, it displayed no significant differences between various methods of administration (P = 0.68; Figure 8).

Discussion

This systematic review and meta-analysis was conducted on a total of seven RCTs involving a comparison of the efficacy of artemether versus quinine in children suffering from cerebral malaria, a common neurological complication caused by malaria [12-18]. To the best of our knowledge, this is the first meta-analysis performed that exclusively assesses the treatment outcomes of children with cerebral malaria, treated using artemether and quinine.

Our analysis demonstrated that artemether had superior performance in the parasite clearance time; however, it showed similar performance to quinine in other metrics, including fever clearance time, coma clearance time, and mortality rate. The typical administration of artemether and quinine includes IM and IV, respectively; this analysis did not discriminate between other modes of administration. The different comparisons included IM artemether versus IV quinine, IM artemether versus IM quinine, and rectal artemether versus IV quinine.

In a systematic review conducted by Kyu et al., no significant differences were noted between the use of artemisinin derivatives as compared to quinine for the treatment of cerebral malaria [19]. Another similar systematic review conducted by Praygod et al. revealed that artemisinin derivatives did not demonstrate any improvements over quinine [20]. Similarly, our analysis illustrated that artemether is comparable to quinine, showing no significant differences between the pre-specified outcomes, with the exception of parasite clearance time, where artemether showed superior performance.

With regard to the several methods of administration explored within this analysis, we included RCTs comparing the efficacy of the IM, IV, and rectal methods of administration of artemether, alongside IM and IV administration of quinine. Although the current treatment guidelines and clinical practice utilize the artemether formulation as an IM-injectable drug [21], rectal administration of artemether is not traditionally used. The analysis demonstrated that the rectal mode of administration showed no significant differences to quinine, except for fever clearance time, where quinine performed significantly better than rectal artemether. The weaker performance of rectal administration for fever clearance time may be due to the lower bioavailability, as well as the increased time needed for the drug to reach systemic circulation, especially when compared to IV administration. Nonetheless, this provides a potential alternative method of administration for the treatment of cerebral malaria in children, as rectal administration of artemether may be advantageous in critically ill patients. This is especially beneficial for treatment within developing countries, as rectal administration provides similarly favorable therapeutic effect, yet is considered an inexpensive form of treatment [22]. Contrastingly, the use of quinine usually involves IV administration for maximal efficacy; however, additional strategies of administration include the IM and rectal methods [23,24]. It was seen that IM quinine performed favorably for coma clearance time as compared to IM artemether. IM quinine is seen as an excellent alternative when IV quinine cannot be used, given that similar studies have outlined the similarity in efficacy of IM and IV quinine [25]. However, due to the lack of included studies comparing rectal artemether with quinine, it is difficult to arrive at a robust conclusion without the need for further studies.

The clinical implications of our results demonstrate the similarity in therapeutic efficacy of both artemether and quinine for the treatment of cerebral malaria in children, with the exception of parasite clearance time. As per the Centers of Disease Control and Prevention (CDC), it is not recommended to initiate treatment until a definitive diagnosis of malaria has been made [26]. The selection of the specific treatment modality depends on the infective Plasmodium species, clinical status of the patient, drug susceptibility, and history of antimalarial use. Uncomplicated cases of malaria can typically be resolved with oral antimalarial treatment; however, cases involving the Plasmodium falciparum species or those that have progressed to severe or cerebral malaria will require aggressive treatment. The CDC recommends the infusion of IV artesunate at 2.4 mg/kg given within intervals of 0, 12, and 24 hours [27], especially in chloroquine-resistant areas. Until recently, artemether and its combinations were the first-line treatment choices according to the WHO. However, with advancing research, it was found that the oil-based artemether had notably erratic and unpredictable absorption when compared to artesunate, leading to the subsequent changes in relevant guidelines [27]. However, artemether is still commonly used in several regions of the world, including Equatorial Guinea, with a prescription rate of nearly 30% (compared to artesunate’s prescription rate of 7%) [28]. With respect to quinine, it is no longer considered the preferred choice for the treatment of malaria, which can be explained by its poor tolerability, complex dosing, and low compliance, alongside its weaker efficacy as compared to other novel anti-malarial drugs on the market such as artemisinin derivatives [29]. However, it is still used in regions with a low stock of alternative anti-malarial agents due to its low availability, and is especially used (and recommended) in nations with increasing artemisinin resistance. To summarize, our analysis mostly showed that quinine had similar performance to artemether and can still be considered an excellent alternative anti-malarial agent for when artemisinin derivatives, such as artemether, cannot be used.

There was considerable heterogeneity observed within our meta-analysis. In the outcome of coma clearance time, there was moderate heterogeneity found, whereas in the outcomes of fever clearance time and parasite clearance time, a high level of heterogeneity was observed. No heterogeneity was noted in the mortality rate. To discover the sources of these heterogeneous outcomes, a sensitivity analysis was performed in cases of high heterogeneity, including the outcomes of fever clearance time and parasite clearance time. This heterogeneity is attributable to the inclusion of studies exploring different methods of administration involved.

This systematic review and meta-analysis is not devoid of limitations. First, this meta-analysis included seven studies that primarily focused on the African continent. The smaller sample size, which exclusively explored limited regions of the world, leads to the underrepresentation of other nations with endemic malaria, and thus may contribute to some heterogeneity and bias within this analysis. Moreover, the included studies showed moderate quality ratings, potentially indicating an overall high level of bias within this analysis, especially with regard to blinding. Therefore, it is encouraged to conduct further, high-quality, randomized studies that may provide improved understanding of the use of artemether or quinine for cerebral malaria in children.

## Conclusions

In conclusion, this systematic review and meta-analysis highlighted the overall similarity between artemether and quinine for the treatment of cerebral malaria in children. However, it was observed that artemether provided superior parasite clearance time when compared to quinine. The seemingly favorable safety profile of artemether reaffirms its superiority over quinine; however, in cases of artemether-resistant strains or in regions where artemisinin derivatives are unavailable, quinine serves as an excellent alternative. Further comprehensive randomized trials, providing comparisons of the safety profiles of both drugs, are highly encouraged in order to establish a reliable conclusion.
